# Intake of green tea inhibited increase of salivary chromogranin A after mental task stress loads

**DOI:** 10.1186/1880-6805-33-20

**Published:** 2014-07-17

**Authors:** Ai Yoto, Sato Murao, Yoriyuki Nakamura, Hidehiko Yokogoshi

**Affiliations:** 1 School of Food and Nutritional Sciences, University of Shizuoka, Shizuoka, Japan; 2 College of Bioscience and Biotechnology, Chubu University, Aichi, Japan

**Keywords:** Green tea, Chromogranin A, Acute stress, Profile of mood states

## Abstract

**Background:**

Green tea has become renowned for its health benefits. In this study, we investigated the anti-stress effect of two kinds of green tea against a mental stress task load.

**Methods:**

Warm water, ordinary green tea (*Sagara*), and shaded white tea, which contains more amino acid components than *Sagara*, were used as test samples in a randomized cross-over design study. Eighteen students (nine male and nine female) participated in three experimental trials on different days at intervals of seven days. Saliva was collected before beverage intake and after performing the mental stress load tasks. Concentration of chromogranin A (CgA) in the saliva was used as an index of autonomic nervous system activity.

**Results:**

CgA level increased after the mental tasks, but intake of green tea inhibited this increase; the anti-stress effect was even greater after consumption of shaded white tea. Intake of shaded white tea also lowered Total Mood Disturbance (TMD) score on the Profile of Mood States (POMS); subjects in this condition tended to perform more calculations in the arithmetic task than those in the warm water treatment condition.

**Conclusions:**

Salivary CgA concentration levels increased after mental stress load tasks, but ingestion of green tea inhibited this increase. This anti-stress effect was larger after the consumption of shaded white tea than after *Sagara*. Shaded white tea intake also lowered TMD score (POMS) and tended to improve performance on an arithmetic task compared to warm water, suggesting that shaded white tea might also improve mood during and after mental stress load.

## Background

Recently, green tea has become renowned for the health benefits of its amino acid components, such as L-theanine (subsequently called theanine) and γ-aminobutyric acid (GABA). Theanine, a popular functional food ingredient, has received attention for its multiple roles in the central nervous system (CNS) and autonomic nervous system (ANS). It has been found to affect dopamine and serotonin concentrations in the brain, thus producing an anxiolytic effect [1,2]. Intake of theanine was also found to increase alpha brain wave activity in humans, which might lead to a relaxed but alert state [3,4]. Kimura reported that theanine reduced heart rate and salivary IgA responses to an acute stress task (arithmetic task), suggesting that it could reduce stress by inhibiting cortical neuron excitation [5].

GABA has also recently been studied for its effects on the CNS and ANS [6-8]. Kanehira *et al*. demonstrated an anti-fatigue effect from GABA ingestion on salivary secretion levels of chromogranin A (CgA) and cortisol following an arithmetic task used as a fatigue load [9]. Nakamura *et al*. also reported the psychological stress-reducing effect of chocolate enriched with GABA (28 mg) on stress induced by an arithmetic task [10]. They found that the GABA-enriched chocolate improved recovery time from a stressful to a normal state, and that in those taking GABA chocolate, CgA values measured after the task were not increased compared to measures before ingestion.

These amino acids are thus likely to underlie the effects of consuming green tea on the brain, including aiding recovery from stress and resistance to depression, promoting a more relaxed state and better mood, and improving sustained attention for mental task performances. Since these psychological and physiological effects of theanine and GABA were observed in studies where the components were administered in a dose-dependent manner, it can be hypothesized that green tea containing more of the amino acids might attenuate stress responses in the ANS even more significantly. However, there is little scientific evidence comparing the anti-stress effects induced by different green tea samples containing different concentrations of amino acid components.

CgA is an acidic glycoprotein with 439 amino acids, occurring in the secretory granules of most neuroendocrine cell types. It is a major protein in adrenal chromaffin cells and adrenergic neurons. Responding to stress, CgA and catecholamines are co-released into the extra-cellular environment. Nakane found a prompt elevation in salivary CgA levels and a delayed increase in salivary cortisol levels when psychosomatic stress was induced by a test involving an oral presentation in front of an audience or a driving situation [11]. Nomura *et al*. also found that salivary CgA concentration depicted an increase during the mental stress tasks and decrease (recovering) during the intermissions, demonstrating the possible candidacy of CgA as a biomarker for a short-term mental workload [12]. These studies suggest that salivary CgA may be a sensitive and promising index for psychosomatic stress.

In this study, we assessed the anti-stress effects of two kinds of green tea - ordinary green tea (*Sagara*), and shaded white tea - on CNS activities in healthy people by measuring salivary CgA. We also evaluated subjects’ Profile of Mood States (POMS) scores and the Visual Analog Scale (VAS) scores as subjective ratings of mental state.

## Methods

### Participants

Eighteen healthy volunteers (9 males, 9 females; ages 23.4 ± 1.85 years) participated in 3 experimental trials at the same time of day with an interval of 7 days between trials. All participants were requested to avoid eating or drinking anything but water for three hours before the start of each trial. The experiment conducted in this study was approved by the research ethics committee of the University of Shizuoka, and was carried out in accordance with the Declaration of Helsinki. All subjects provided informed consent after receiving an explanation of the experimental protocol.

### Procedure

**Figure 1:**
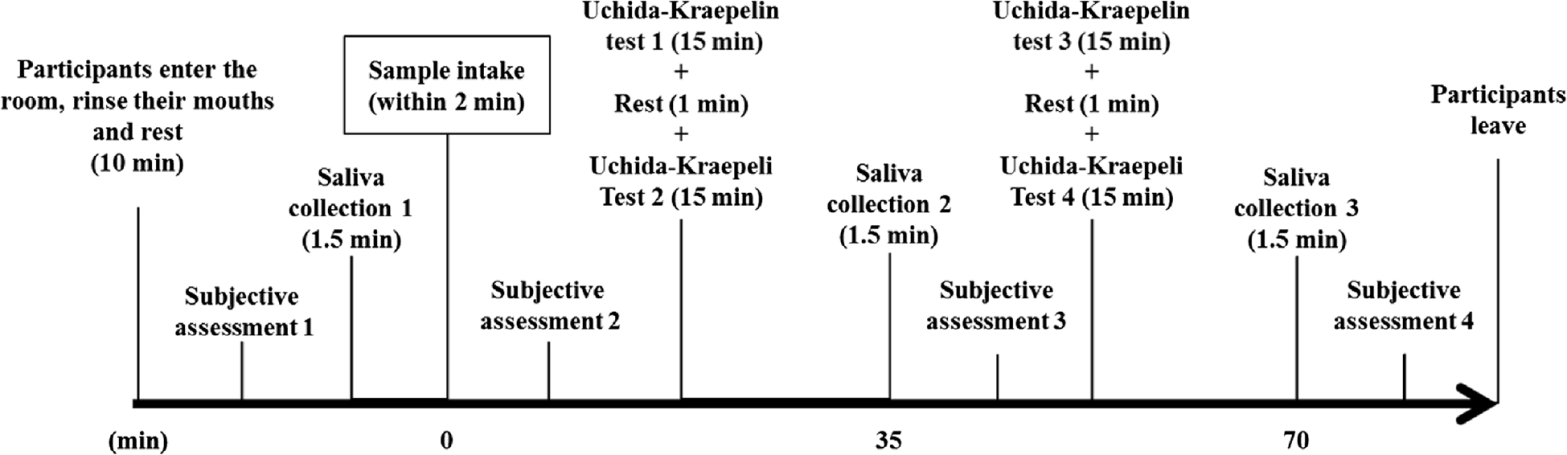
**Procedures used in this study.** Saliva collection and subjective assessments, including Profile of Mood States (POMS) and Visual Analog Scale (VAS), were performed before, and again 30 minutes and 60 minutes after ingestion of sample. An additional subjective assessment was also completed immediately after sample intake.

Figure 1 shows the experimental procedure. All participants were required to complete sessions on a total of 3 study days with 1-week intervals between experiment days; total session time per day was about 1.5 hours. Experimental sessions were held from 09:00 to 10:30 in July. The room temperature was 25.8°C. On the day of the experiment, when participants entered the room, they were seated and asked to rest for ten minutes. During the resting time, participants rinsed their mouths with a cup of water. After resting, the first subjective assessment and the first saliva collection were completed to obtain baseline data. Participants then drank one of the beverage samples, followed by the second subjective assessment. They then began the 31-minute mental stress load task session (2 Uchida-Kraepelin (U-K) tests with a 1-minute rest). The stress load task was followed by a measurement session that included the second saliva collection and third subjective assessment. At about 35 minutes after sample ingestion, participants performed another 31-minute mental stress load task session. Finally, a third saliva collection and fourth subjective assessment were completed.

### Treatment

A cross-over, single blind, randomized design was used in this study. Three separate trials were performed in which the participants ingested one of three beverage samples (water as a control and two kinds of green tea: *Sagara* and shaded white tea) each day. *Sagara* and shaded white tea were made from the Japanese *Yabukita* tea plant by a different process. Shaded white tea was specifically grown in perfectly blocked sunlight before plucking. Tea samples were prepared using each of their ordinary brewing method for serving these different kinds of green tea [13]. *Sagara* was extracted with 900 ml of 90°C hot water for 30 seconds from 20 g of *Sagara* tealeaf; shaded white tea was extracted with 900 ml of 70°C hot water for 2 minutes from 30 g of the tealeaf. After extraction, tealeaves were removed and 250 ml of the tea sample was cooled to 25°C and then poured into a paper cup for subjects to drink. The warm water used as the control sample was prepared by cooling hot water to 25°C.

Levels of amino acid components in the two tea samples were measured in sulfosalicylic acid deproteinized samples by Ion Exchange Chromatography using a JLC-500/V AminoTac™ Amino Acid Analyzer (JEOL Ltd., Tokyo, Japan) [14]. L-theanine (Suntheanine, Taiyo Kagaku Co., Ltd, Mie, Japan) and the amino acids mixture standard solution (Wako Pure Chemical Industries, Ltd., Osaka, Japan) were used as reference amino acid standards. The concentrations of caffeine and catechins in the two tea samples were measured with the Acquity UPLC System (Waters Corp., Milford, MA, US), using Acquity BEH Shield RP18 column with 2.1 × 100 mm inner diameter and 1.7 μm particle size [15].

### Stress load task

The U-K test was used as the stress load task. The test, a questionnaire modified from Kraepelin’s arithmetic test and developed by Uchida [16], is widely used as a mental stressor [17-19]. In this study, participants were given a pre-printed paper containing 15 lines of random, single-digit, horizontally aligned numbers, and asked to perform calculations as quickly and accurately as possible for 15 minutes. After a one-minute rest period, the test was repeated, so that one stress-load session lasted about thirty-one minutes. Two sessions were performed in each trial. The average number of answers and percentage of correct answers for each test were used as indices of task performance.

### Subjective assessment

The POMS and VAS, used for subjective ratings on mood state, were completed before ingestion (baseline data), immediately after ingestion, and again after the two mental task sessions were completed.

We used a short version of POMS to assess distinct affective mood states. The POMS is a popular tool used widely among psychologists and scientists from many fields. Six identifiable mood or affective states were scored from the POMS: Tension-Anxiety, Depression-Dejection, Anger-Hostility, Vigor-Activity, Fatigue-Inertia, and Confusion-Bewilderment. Total Mood Disturbance (TMD) was then calculated from these six scores. A higher TMD score indicated a more negative affective state; that is, positive changes in mood were reflected by negative changes to TMD scores. All of the scores were used for analysis in this study.

Participants’ feelings of pressure, drowsiness, stress, relaxation, fatigue, reassurance and tension were assessed using VAS. A continuous, 10-cm VAS rating scale was used, with the 0 end point representing ‘do not feel’ and the 10 end point indicating ‘strongly feel’. Subjects were asked to make a mark on the line that represented their mood at the time. Immediately after ingestion, two more scales were also included in the VAS asking about preference and familiar experience with the drink samples.

### Saliva collection and CgA measurements

Saliva was collected in Salivette tubes (Sarstedt, Nümbrecht, Germany) and centrifuged at 3,000 rpm. The supernatant was transferred into Eppendorf tubes and frozen at -80°C for later measurement. Concentration of CgA in the saliva samples was subsequently determined by ELISA (Yanaihara Institute Inc., Shizuoka, Japan). At the same time, total protein was measured with the dye-binding assay of Bradford (Bio-Rad Protein Assay, Hercules, CA, US). Measured values of CgA were divided by the protein concentration and used for further analysis.

### Statistical analysis

Data were analyzed using IBM SPSS Statistics version 19 (Chicago, IL, USA). All data are expressed as the mean ± standard error, and *P* < 0.05 was considered significant.

#### ***Time effects***

Across the four time points (before intake, immediately after intake, after U-K test two, and after U-K test four), the effect of time in POMS scores were analyzed by Friedman tests and Wilcoxon signed rank tests with Bonferroni correction.

Kruskal-Wallis tests and Mann-Whitney *U*-tests with Bonferroni correction were conducted to detect differences of CgA concentrations among the four time points.

#### ***Sample effects***

Repeated measure one-way analysis of variance (ANOVA) and Bonferroni *post hoc* tests were performed to detect differences of task performance among the three sample conditions (warm water, *Sagara* and shaded white tea).

Changes of VAS score regarding mood state and changes of POMS score from baseline were analyzed by Kruskal-Wallis tests.

VAS scores immediately after intakes asking about preference and familiar experience with the drink samples and the concentrations of CgA were analyzed by Friedman tests and Wilcoxon signed rank tests with Bonferroni correction.

## Results

### Determination of main functional tea components

**Figure 2:**
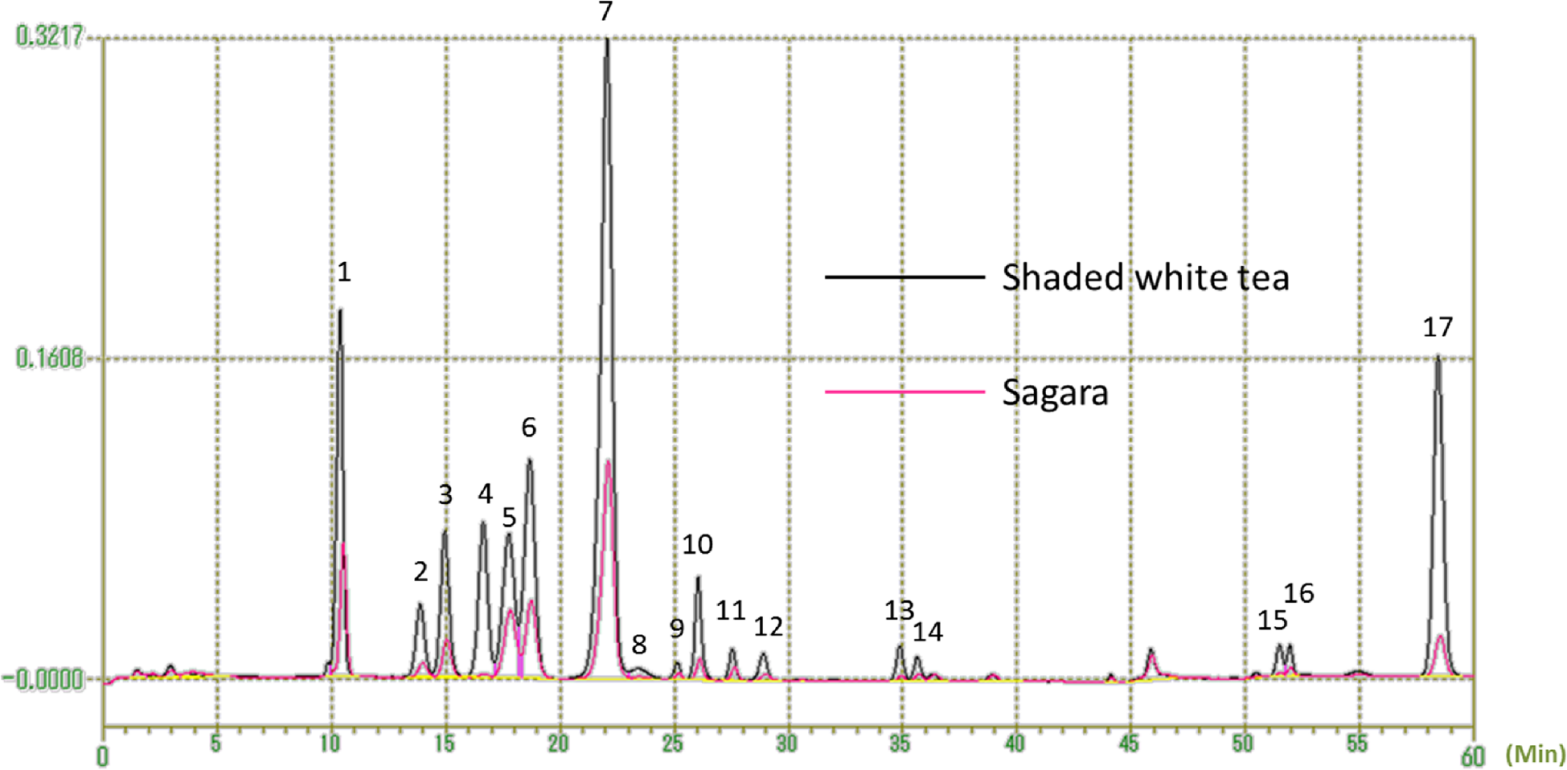
**Levels of amino acid components in the two test tea samples (*Sagara *- pink line; shaded white tea - black line) measured with the Amino Acid Analyzer (JEOL JLC-500/V AminoTac™, Japan).** Peaks with numbers at the top represent 17 amino acid components. Results reveal an approximately three-fold or higher concentration in shaded white tea. Peak number: 1 aspartic acid, 2 threonine, 3 serine, 4 asparagine, 5 glutamic acid, 6 glutamine, 7 theanine, 8 alpha-aminoadipic acid, 9 glycine, 10 alanine, 11 valine, 12 cystine, 13 isoleucine, 14 leucine, 15 histidine, 16 lysine, 17 arginine.

Figure 2 shows test results of amino acids for *Sagara* (pink line) and shaded white tea (black line). The peaks with numbers at the top represent 17 amino acid components. As may be seen in the figure, concentration of these components was approximately three-fold or even higher in the shaded white tea than in the *Sagara*.

The concentrations of caffeine and catechins in the two tea samples were 192 mg and 223 mg, respectively, in the shaded white tea, and 87 mg and 304 mg, respectively in the *Sagara*. Thus, the level of caffeine was higher and the level of catechins lower in the shaded white tea than in the *Sagara*.

The above concentrations of each amino acid, caffeine and catechins of the two green tea samples are listed in Table 1.

**Table 1:** The concentrations of amino acid, caffeine and catechins in the green tea samples

		***Sagara***	**Shaded white tea**
Amino acids (nmol/ml)	aspartic acid	118.52	339.07
	threonine	16.82	88.25
	serine	45.68	175.81
	asparagine	11.22	487.45
	glutamic acid	122.30	256.41
	glutamine	157.13	428.93
	theanine	401.12	1198.58
	alpha-aminoadipic acid	6.94	32.65
	glycine	5.71	15.01
	alanine	ND	91.05
	valine	23.38	55.21
	cystine	3.57	15.24
	isoleucine	5.15	38.29
	leucine	7.43	25.52
	gamma-aminobutyric acid	5.68	9.64
	histidine	3.27	31.78
	lysine	7.10	25.87
	arginine	70.71	550.54
Caffeine and catechins (mg/250 ml)	caffeine	87.47	191.60
	catechins	303.78	223.08

ND: not detected.

### Task performance

**Figure 3:**
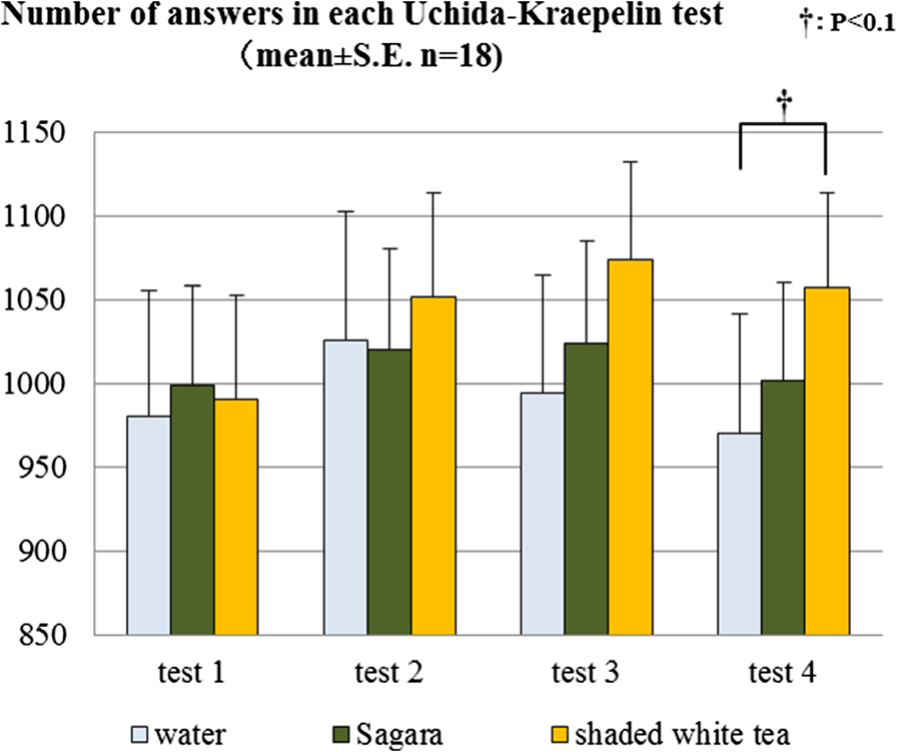
Average numbers of answers in each Uchida-Kraepelin (U-K) test, and results of the Bonferroni multiple comparisons.

Figure 3 presents the average number of answers in each U-K test. The results of the repeated measure one way ANOVA showed that the number of answers in the fourth round of the test appeared to be affected by the sample treatments (F(2,34) = 3.168, *P* < 0.1). The Bonferroni multiple comparisons showed that participants tended to perform more calculations after ingesting the shaded white tea than after ingesting warm water (*P* < 0.1).

### VAS

**Figure 4:**
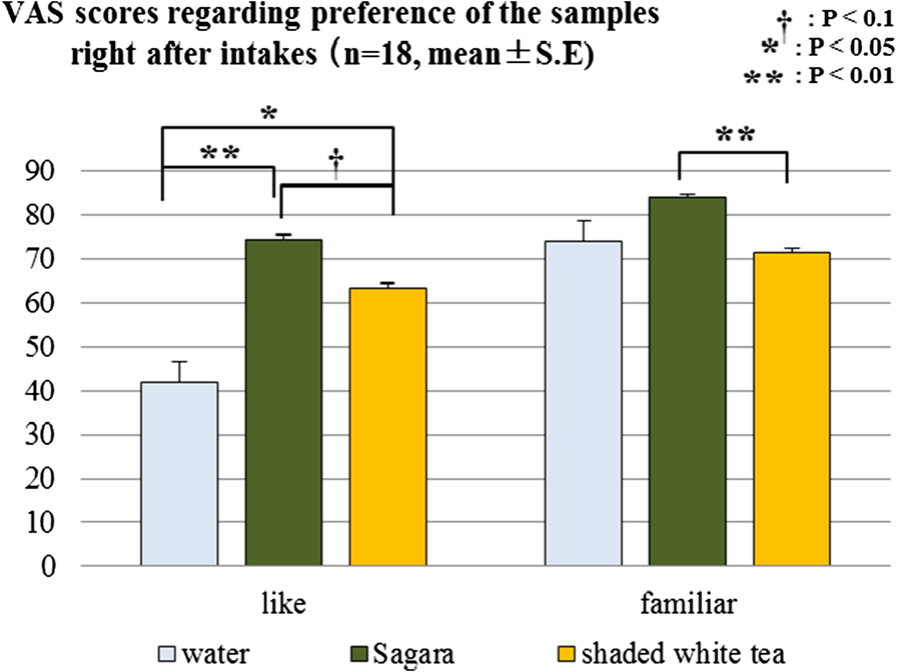
Visual Analog Scale (VAS) scores of preference and familiar experience regarding the drink samples, and the comparisons using Wilcoxon signed rank tests with Bonferroni correction.

Changes from baseline in the scores for items regarding mood state were analyzed by Kruskal-Wallis tests, but no significant sample effect could be found (data not shown). On the other hand, VAS scores taken immediately after sample ingestion showed that feelings of preference and familiar experience regarding the drink samples were different among the three conditions by Friedman tests (*P* < 0.01, 0.05). As Figure 4 shows, the results of Wilcoxon signed rank tests with Bonferroni correction showed that the ‘like’ scores for *Sagara* were significantly higher than those for warm water, and also tended to be higher than those for shade white tea (*P* < 0.01/3, 0.1/3). Shaded white tea also had a higher ‘like’ score than warm water (*P* < 0.05/3). With regards to familiar experience, *Sagara* had a higher score than shaded white tea (*P* < 0.05/3).

### POMS

**Figure 5:**
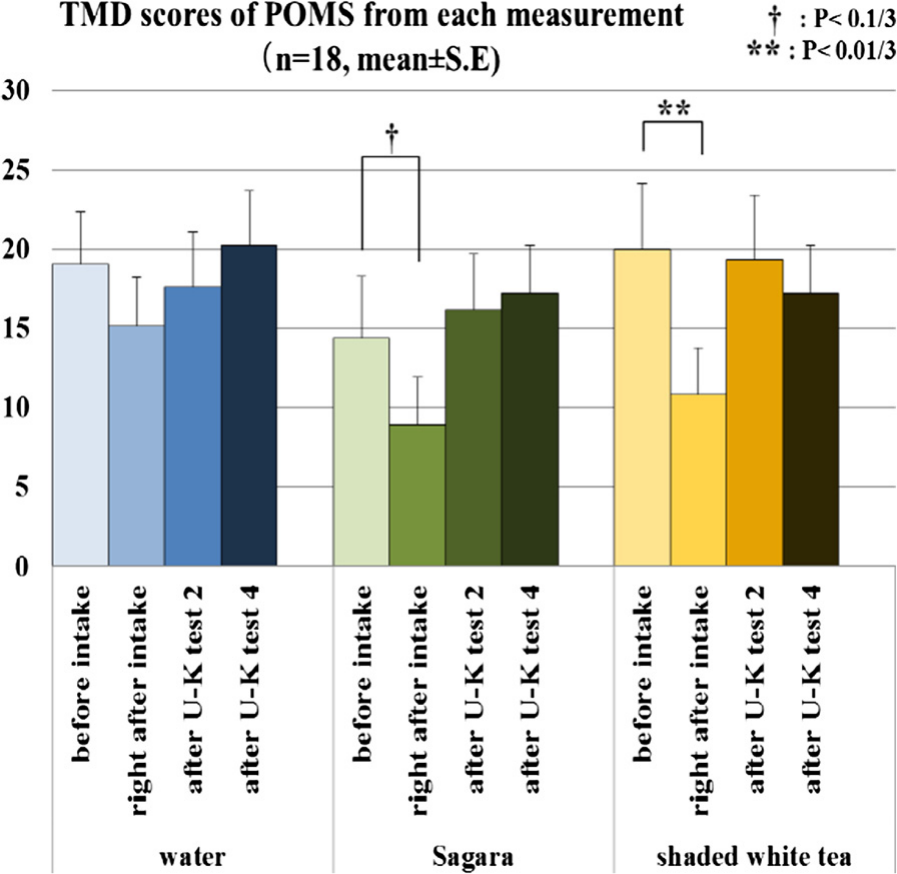
Total Mood Disturbance (TMD) scores of Profile of Mood States (POMS), and the results of comparisons by Wilcoxon signed rank tests with Bonferroni correction.

The effect of time in the POMS scores by Friedman tests showed differences in TMD scores for the two tea treatment conditions (*P* < 0.01). Wilcoxon signed rank tests indicated that the ingestion of shaded white tea decreased the TMD score significantly compared with the baseline evaluation (*P* < 0.01/3); *Sagara* also tended to decrease the TMD score, but to a lesser degree than shaded white tea (*P* < 0.1/3, Figure 5). Kruskal-Wallis tests were also performed to detect differences among sample treatments using changes from the baseline in the scores of TMD and other POMS scores, but no significant result was found.

### CgA

**Figure 6:**
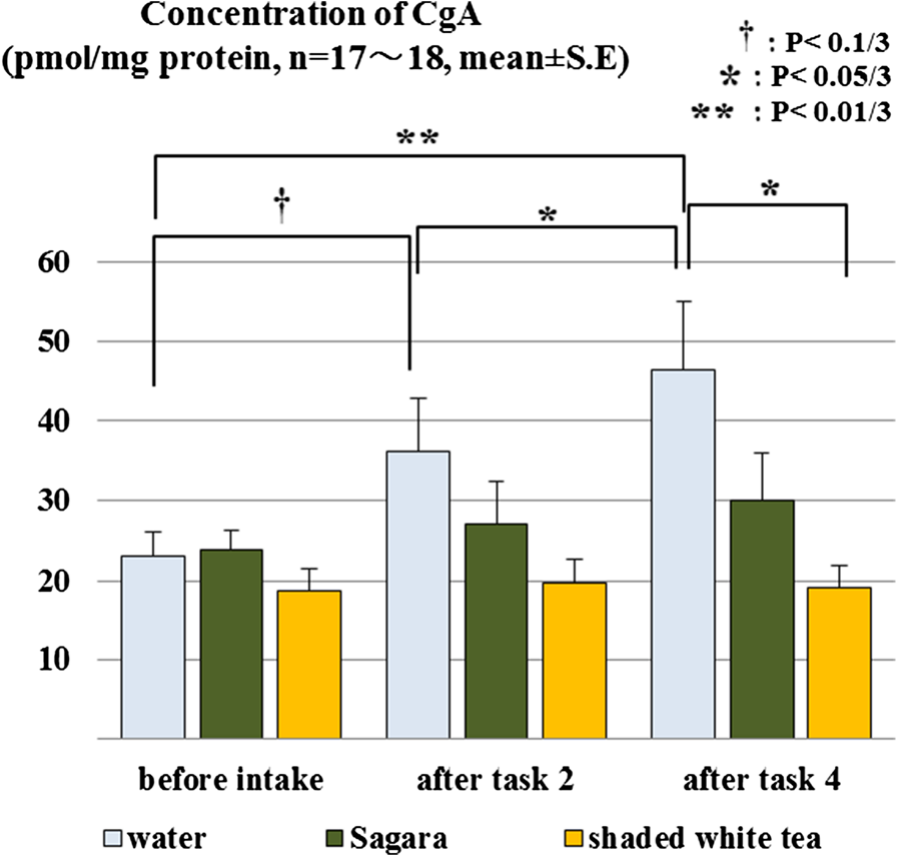
**Salivary chromogranin A (CgA) levels at each time point in each treatment condition.** Comparisons were performed using Mann-Whitney *U*-tests with Bonferroni correction.

Across the time course, concentrations of CgA differed only in the warm water treatment condition (*P* < 0.01). Results of the Mann-Whitney *U*-tests showed that CgA concentrations increased significantly after mental stress task sessions (*P* < 0.1/3, 0.01/3 after U-K test 2 and test 4, compared with before ingestion, respectively, *P* < 0.05/3 comparing the concentration after test 4 with after test 2, Figure 6).

Among the 3 drink conditions, differences were found after U-K test 4 by Kruskal-Wallis tests (*P* < 0.05). Mann-Whitney *U*-tests revealed that CgA concentration after U-K test 4 in the shaded white tea treatment condition was lower than in the warm water treatment condition (*P* < 0.05/3).

## Discussion

The results of CgA tests showed that U-K tests increased salivary CgA levels significantly only in the warm water condition. That is, the two tea treatments attenuated CgA increase induced by the stress of performing the U-K tests, demonstrating the anti-stress effects of the teas. Green tea contains many chemicals that are reported to affect the human cardiovascular system and the brain, including amino acids, theanine and GABA. Caffeine and catechins also have been reported to improve mood and reduce anxiety or self-rated stress [20,21].

As expected, participants who ingested shaded white tea had significantly lower CgA levels after completing all U-K tests than those who ingested water. There was not, however, a significant difference found between *Sagara* and warm water. In other words, the anti-stress effect of consuming shaded white tea was considerably larger than that of the ordinary green tea, *Sagara*. Quinlan *et al*. reported that tea and coffee produced mild autonomic stimulation and an elevation in mood but there were no effects of caffeine dose [22]. They concluded that caffeine could exert dose-dependent effects on a number of acute autonomic responses, meanwhile caffeine level was not an important factor, and factors besides caffeine might contribute to those acute effects. Thus, the higher dose of caffeine in shaded white tea may not be the only component that caused the different results in CgA levels between the two tea samples.

As shown in Figure 2 and Table 1, levels of amino acids, including theanine, were substantially higher in shaded white tea than in *Sagara*. Detected amounts of GABA and theanine were 0.25 mg and 52 mg, respectively, in shaded white tea, while they were 0.15 mg and 17 mg in *Sagara*. Knowing that most human mood studies have used higher doses of theanine (for example, 200 mg in Juneja *et al*. [3] and Higashiyama *et al*. [23]; and 250 mg in Gomez-Ramirez *et al*. [24]), the low dose of 52 mg in shaded white tea in this study might have been insufficient. However, one previous study on the effect of theanine consumption on human brain alpha activity found significantly greater and increasing alpha activity after ingestion of 50 mg theanine compared with placebo [25]. Alpha activity is indicative of a state of wakeful relaxation, and is associated with decreased anxiety and improved performance under stress [26,27]. At the same time, previous studies have indicated that theanine interacted with caffeine, and the combination promoted better effects than either alone [28]. Thus the mixture of theanine and caffeine in shaded white tea may have potentiated the anti-stress effect beyond that seen in studies using 50 mg theanine alone.

Besides the theanine and GABA, other amino acid components that showed much higher content levels in shaded white tea than in *Sagara* also deserve attention. These include an asparagine level forty-three times greater, a twice greater glutamic acid level, alanine (none in *Sagara*), an isoleucine seven times greater and an arginine level eight times greater. It is still unclear whether these components play important roles in improving mood or reducing stress. Further studies of these isolated amino acid components are necessary to confirm their physical and psychological effects. Nevertheless, green tea has been traditionally used as an herbal medicine since ancient times, and, in general, herbal medicines are complex mixtures of different compounds that often act in a synergistic fashion to exert their full beneficial effect. From this point of view, it seems plausible to consider that the high content of the amino acid compounds mixed with higher content level of caffeine contributed to the larger anti-stress effect of shaded white tea in this study.

It may be that the anti-stress effect promotes a more relaxed state that improves sustained attention, thus leading to the improved performance on the U-K tests. Participants tended to complete more calculations after ingesting shaded white tea than after ingesting warm water. Among the large body of literature on the performance effects of caffeine and catechins, amino acid components such as theanine have also been reported to have positive effects on brain activity such as memory and learning ability [3,29]. Additionally, an *in vitro* study reported that glutamic acid in green tea increased electrical hippocampal activity in rats; such activity is commonly taken as representative for enhancement of spatial and time-dependent memory [30].

Finally, even though the results of the VAS indicated that shaded white tea elicited lower scores in terms of preference and familiar experience compared with the ordinary green tea *Sagara*, the intake of shaded white tea significantly decreased the TMD score measured immediately after ingestion compared with the baseline evaluation; meanwhile, ingestion of *Sagara* showed only a trend of this effect. Needless to say, there was no similar effect from warm water intake. We presume that this TMD score decrease came from the influence of the odor of the green tea. Our previous study showed that smelling shaded white tea increased the band power of alpha and beta in the frontal and occipital regions and suppressed the decrease of Vigor-Activity in POMS after mental tasks [31]. The mechanism might be that one of the main odor components of green tea stimulated the release of dopamine, which regulates brain functions, mood and attention in rats, and induced changes of event-related potential P-300 and perceived pleasure in human studies [32,33].

The limitations of our study are that only young Japanese students participated in this study, and only the concentration of the salivary CgA was used as the index for ANS activity. Further study is needed to explore the anti-stress effects of consuming shaded white tea or other amino acid-rich green tea using participants across all age groups and from other geographical regions by measuring more ANS or/and CNS activities.

## Conclusions

Salivary CgA concentration levels increased after mental stress load tasks, but ingestion of green tea inhibited this increase. This anti-stress effect was larger after the consumption of shaded white tea than after *Sagara*. Shaded white tea intake also lowered TMD score (POMS) and tended to improve performance on an arithmetic task compared to warm water, suggesting that shaded white tea might also improve mood during and after mental stress load.

## Abbreviations

ANOVA: analysis of variance; ANS: autonomic nervous system; CgA: chromogranin A; CNS: central nervous system; EEG: electroencephalogram; GABA: γ-aminobutyric acid; Ig: immunoglobulin; POMS: Profile of Mood States; TMD: Total Mood Disturbance; U-K: Uchida-Kraepelin; VAS: Visual Analog Scales.

## Competing interests

The authors declare that they have no competing interests.

## Authors’ contributions

AY conceived and designed the study, performed the experiments and the statistical analysis, and drafted the manuscript. SM helped to carry out the experiments and to perform data analysis. YN and HY conceived of the study, participated in its design and coordination, and helped to draft the manuscript. All authors have read and approved the final manuscript.
